# Prognostic Value of Galectin-3 after Left Atrial Appendage Occlusion for Predicting Peri-Device Leakage

**DOI:** 10.3390/ijms242316802

**Published:** 2023-11-27

**Authors:** Franz Haertel, Paul Lustermann, Ali Hamadanchi, Katja Gruen, Jurgen Bogoviku, Pawel Aftanski, Julian Westphal, Laura Baez, Marcus Franz, P. Christian Schulze, Sven Moebius-Winkler

**Affiliations:** Department of Internal Medicine I, Cardiology, University Hospital Jena, Am Klinikum 1, 07747 Jena, Germany

**Keywords:** LAA occluder implantation, fibrosis, atrial fibrillation, anticoagulation, left atrial appendage, TEE, galectin-3

## Abstract

Echocardiographic detection of residual peri-device leakage (PDL) after percutaneous left atrial appendage occlusion (LAAO) is crucial for managing anticoagulation. Galectin-3, a protein involved in tissue–foreign body interactions, may hold significance in understanding PDL and cardiac tissue remodeling after LAAO. This study aimed to analyze galectin-3 serum levels in relation to PDL using a novel echo-morphological classification. LAAO eligible patients were included in the study. Galectin-3 serum levels were measured before LAAO, at 45 days (45D), and at 6 months (6M) after the procedure. Transesophageal echocardiography was used to assess LAAO success. A new echo-morphological classification categorized the degree of LAAO into three different types (A: homogenous echodensity, indicating completely thrombosed device; B: inhomogeneous echolucencies (<50% of device); and C: partially thrombosed device with echolucencies > 50%). Among 47 patients, complete LAAO was achieved in 60% after 45D and in 74% after 6M. We observed a significant increase and distribution of serum levels of galectin-3 [ng/mL] after 45D among the three types (baseline: 13.1 ± 5.8 ng/mL; 45D: 16.3 ± 7.2 ng/mL (Type A) vs. 19.2 ± 8.6 ng/mL (Type B) vs. 25.8 ± 9.4 ng/mL (Type C); *p* = 0.031), followed by a drop in galectin-3 for Types A and B after 6M toward and below the baseline levels (6M: 8.9 ± 3.1 ng/mL (Type A) vs. 12.4 ± 5.5 ng/mL (Type B)), whereas Type C persisted in showing elevated galectin-3 levels compared to all other types (6M: 17.5 ± 4.5 ng/mL (Type C); *p* < 0.01). Increased galectin-3 serum levels after LAAO likely reflect the transition from thrombus formation to fibrotic scar development in the LAA lumen. Successful occlusion is associated with a time-restricted decrease in galectin-3 levels after 6 months, while relevant PDL leads to persistently elevated levels, making galectin-3 a potential predictor of occlusion success.

## 1. Introduction

Major bleeding occurs in approximately 5% of patients with non-valvular atrial fibrillation (AF) taking oral anticoagulants, as reported in current guidelines [1]. Among those bleedings, intracerebral and gastrointestinal manifestations represent the most common forms [2]. Thus, approximately 30–40% of AF patients who meet the criteria for oral anticoagulation are not treated due to the relative or absolute risks of bleeding [3,4,5].

Left atrial appendage (LAA) occlusion (LAAO) has been developed for the prevention of thromboembolic events, including stroke, as an alternative for patients with contraindications for permanent therapeutic anticoagulation and a relevant risk of stroke represented by a CHA_2_DS_2_VASc Score > 1 [6].

After a documented successful occlusion of the LAA, both surgically or via an interventional procedure, oral anticoagulation can be permanently stopped, and the efficacy and safety of this procedure has been demonstrated in three large randomized studies [4,7,8]

Despite successful interventional LAAO approaches at the time of occluder implantation, incomplete LAAO or peri-device leaks (PDLs) can develop over time [8,9,10,11,12]. This phenomenon, occurring in up to 36% of patients, results from remodeling of the LAA tissue surrounding the device and is additionally triggered by the varying LAA size and shape depending on heart rhythm post implantation (AF versus sinus rhythm) and the filling status of the left atrium (LA) [8,9,10,11,12].

It is uncertain whether these PDLs are clinically significant, and there is currently no consensus about how to manage these patients [11]. The occluded LAA has minimal contraction and is particularly prone to local stasis and, thus, thrombus formation [13,14]

Observations from early trials suggested no increase in stroke risk among patients with persistent PDLs [11]. However, in the case of leaks of 5 mm or greater with low blood flow velocities, it may be necessary for clinicians to continue oral anticoagulation, if tolerated, or close the leak percutaneously [11]. However, a large registry study by Alkhouli et al. including > 50,000 patients demonstrated that small leaks (0–5 mm), in particular, were associated with a modestly higher incidence of thromboembolic and bleeding events after LAAO, and large leaks (>5 mm) were not associated with adverse events, even though a higher proportion of these patients were put on continued anticoagulation [15]. It should be noted that only a small proportion of patients, approximately 330 out of 50,000, had significant leaks [15]. As a result, the significance of this finding is controversial.

Evaluation of successful LAAO is routinely performed during follow-up using transesophageal echocardiography (TEE) as the standard imaging modality with defined criteria [16].

Our study group recently proposed a novel echocardiographic classification for the prediction of PDLs based on morphology and the amount of echodensity inside the devices, which features three types [17].

Considering that active remodeling of the LAA occurs during the replacement of a thrombus by fibrotic scar tissue inside the LAA after occlusion, a question arises regarding if associated biomarkers known as circulating reflectors of organ fibrosis might potentially serve as non-invasive surveillance tools predicting occlusion success.

One interesting biomarker in this context is galectin-3, which has been widely studied in cardiovascular diseases [18]. In brief, this molecule enhances inflammation-mediated fibrosis induction and is, among others, extensively expressed in and secreted by activated fibro-/myofibroblasts in the heart [19,20]. Besides its role in a variety of other cardiovascular disorders, galectin-3 plays a crucial role in the progression of AF-associated atrial remodeling, which itself perpetuates AF [21]. Regarding the LAA, which is a structure of interest in our study, circulating galectin-3 has been shown to be linked to LAA remodeling and thrombus formation in AF patients [22]. Also, the possible relationship between galectin-3 serum levels and LAAO has been addressed in a sub-analysis of the PRAGUE-17 trial. Here, no relevant differences could be shown when comparing the 6-month follow-up with the baseline [23].

Considering these findings, the aim of our current study was to analyze galectin-3 serum levels before LAAO as well as during structured follow-up at the timepoints of 45 days and 6 months after occlusion. Results were related to the occurrence of PDLs and, in particular, to the three types of the novel echo-morphological classification described recently by our group [17].

## 2. Results

### 2.1. Patient Cohort

We included 47 patients in the present study. Table 1 presents the baseline demographic characteristics and morphology-specific characteristics of the LAAs at the time-point of the intervention. No patient died or was in need of rehospitalization due to bleeding complications during the follow-up period. One pericardial effusion occurred immediately after the procedure while in hospital. Additional cardiac devices, such as valves or pacemakers, were not implanted in any of the patients during the follow-up period.

### 2.2. Echocardiographic Morphology after LAAO

The mean baseline LAA diameter before LAAO was determined by TEE to be 21.7 ± 3.6 mm. During the procedure, devices with a mean diameter of 26.4 ± 3.9 mm were implanted. The corresponding compression rate at baseline measured 22.2 ± 8.5%. Complete LAAO (without any residual flow) was achieved in 60% (28 patients) of patients after 45 days and in 74% (35 patients) of patients after 6 months.

**Figure 1:**
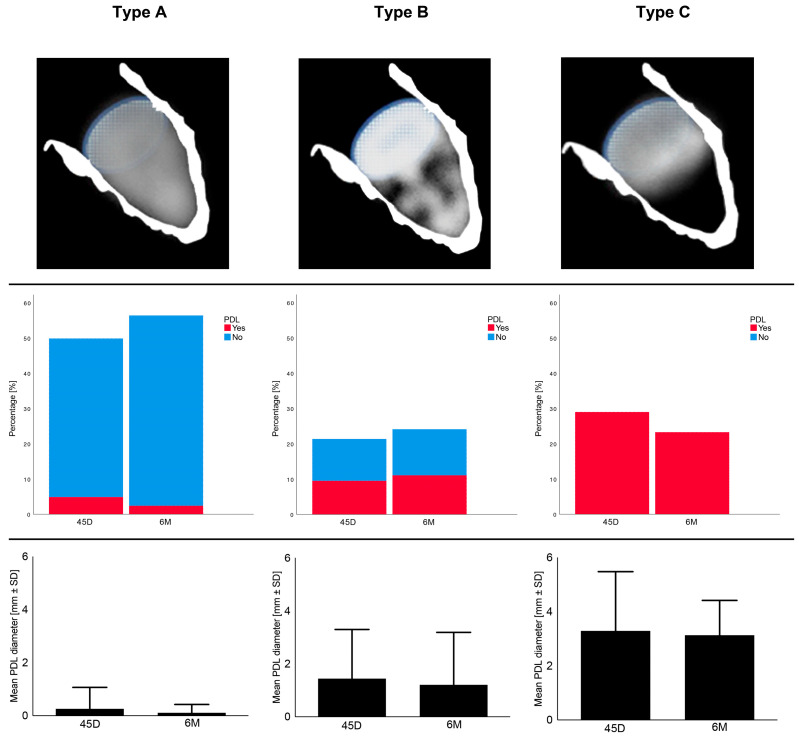
Echocardiographic morphology and features 45 days (45D) and 6 months (6M) after LAAO regarding Types A–C; SD = standard deviation, PDL = peri-device leakage.

Applying the echocardiographic morphology classification according to Hamadanchi et al. [17], we observed the frequencies of PDL and the respective PDL diameters for each type during follow-up appointments, as shown in Figure 1. It becomes clear that Type A is associated with less PDL occurrence and smaller PDL diameters as opposed to Types B and C.

### 2.3. Dynamics of Galectin-3

**Figure 2:**
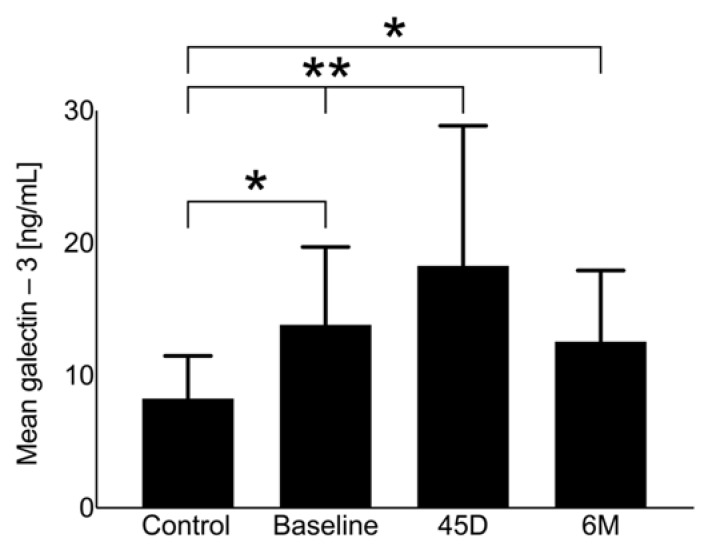
Galectin-3 levels of the study population at baseline, 45 days (45D), and 6 months (6M) following LAAO in comparison with the control group, ** *p* < 0.05, * *p* > 0.05.

Galectin-3 at baseline does not differ significantly in comparison with the control group without AF and, therefore, there is no indication for LAAO (Figure 2). Figure 2 demonstrates the changes during the follow-up period until 6 months after LAAO, showing a peak in galectin-3 around 45 days (control group vs. 45D: *p* < 0.01; baseline vs. 45D: *p* = 0.014) and a decrease to levels near baseline at 6 months. Galectin-3 levels did not show a significant difference regarding the type of AF at baseline (paroxysmal AF: 11.7 ± 5.4 ng/mL vs. permanent AF: 12.1 ± 6.3 ng/mL; *p* = 0.45).

### 2.4. Association of Galectin-3 with Echocardiographic Morphology

**Figure 3:**
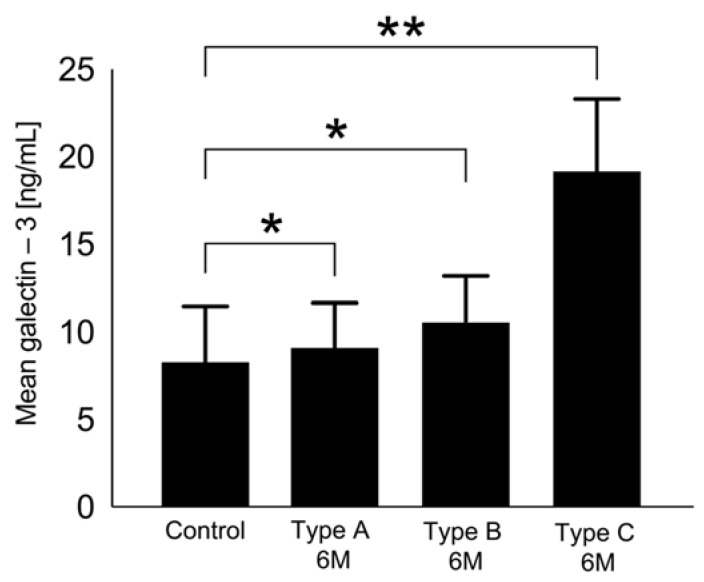
Galectin-3 levels 6 months after LAAO regarding Types A–C in comparison with the control group, ** *p* < 0.05, * *p* > 0.05.

**Figure 4:**
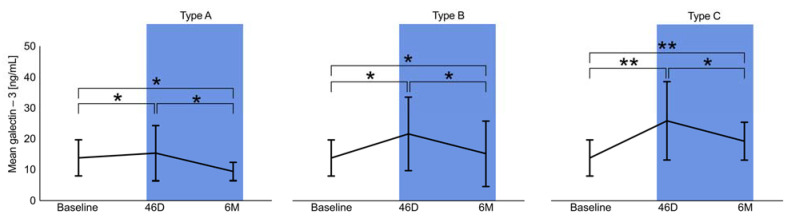
Dynamics of galectin-3 levels at baseline, 45 days (45D), and 6 months (6M) regarding Types A–C, ** *p* < 0.05, * *p* > 0.05.

The dynamics of galectin-3 serum levels during the follow-up period showed different patterns in association with the morphology after LAAO (Types A–C). Type A (full thrombosis within the occluder) revealed the weakest dynamics and even regressed after 6 months to below baseline (Figure 3 and Figure 4, respectively). Depending on the degree of delayed thrombosis within the occluder, elevated galectin-3 levels beyond the 45D follow-up period were recorded (Figure 3 and Figure 4). Correlation analysis showed a significant trend between galectin-3 and mean PDL diameter (0.51; *p* = 0.016), and a PDL diameter of >2 mm was associated with significantly higher galectin-3 levels (45D: 23.1 ± 12.4 ng/mL vs. 15.1 ± 7.9 ng/mL, *p* = 0.03; 6M: 16.6 ± 4.4 ng/mL vs. 9.7 ± 4.1 ng/mL, *p* = 0.02).

### 2.5. Prognostic Value of Galectin-3 for Type C

**Figure 5:**
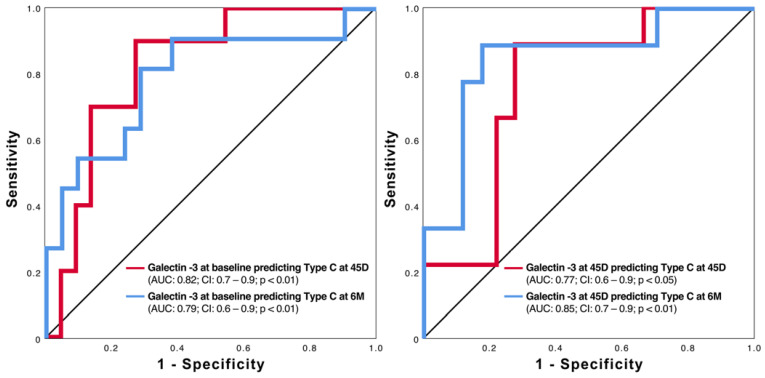
ROC curves of galectin-3 at baseline (**left side**) and 45 days (**right side**) in predicting Type C at 45 days (45D) and 6 months (6M) after LAAO.

**Figure 6:**
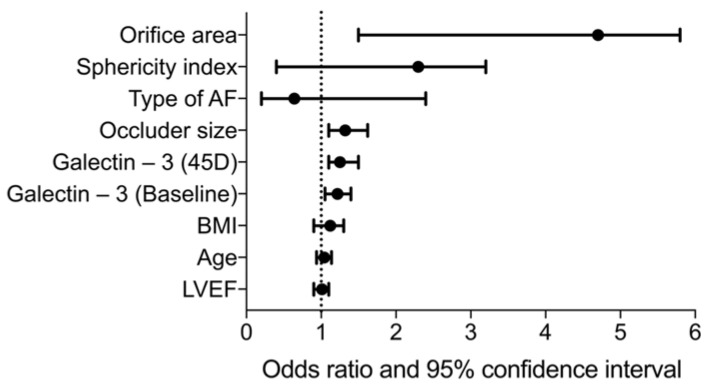
Forest plot showing the odds ratios of selected clinical parameters, echocardiographic LAA features, and galectin-3 (baseline and at 45 days). AF = atrial fibrillation; BMI = body mass index; LVEF = left ventricular ejection fraction.

Using galectin-3 levels at baseline and at the 45-day follow-up, it is possible to predict the clinically relevant Type C morphology after LAAO (Figure 5). Further evaluation using binary linear regression reveals relevant odds ratios for galectin-3 at baseline and after 45 days (Figure 6). However, as shown in Figure 6, occluder size and orifice area outperform the two galectin-3 parameters.

### 2.6. Cut-Off Values for Galectin-3 Predicting Type C

On the basis of the Youden’s index and the reported AUCs, cut-off values for the presence of Type C were calculated and are summarized in Table 2 together with the respective sensitivity and specificity. Values of galectin-3 lower than 5.2 ng/mL are considered to indicate the absence of significant PDL, while levels higher than 17.1 ng/mL are strongly associated with significant leakage.

## 3. Discussion

Peri-device leaks after interventional LAA occlusion are common and associated with increased numbers of strokes. Therefore, detection of PDL after LAAO is crucial and usually carried out via invasive methods like TEE or contrast-enhanced CT scans. Measuring molecular markers of fibrosis like galectin-3 might be useful for developing a biomarker capable of verifying complete LAA closure. So far, this biomarker has not been under investigation in that regard. Thus, the current study was performed to elucidate the potential value and feasibility of galectin-3 as a non-invasive surveillance tool to evaluate LAAO success.

In brief, our current study revealed that tissue fibrosis is primarily reflected by a peak in galectin-3 serum levels during the weeks following LAAO. This finding highlights the occlusion concept, as galectin-3 returns to physiological levels similar to those of the control group. This phenomenon was demonstrated approximately 6 months after LAAO. However, this result was only achieved in complete LAAO. PDL seems to maintain active tissue and extracellular matrix (ECM) remodeling. Therefore, the ongoing expression of galectin-3 can be found to be relevantly elevated in these patients.

Besides thrombus formation in an occluded LAA, LAAO means implanting foreign material into the left atrial appendage resulting in an inflammatory and fibrotic tissue reaction. Within the interface between the LAA tissue and the occlusion device, immune cells become attracted to the interface and fibroblasts are activated, including their transdifferentiation to myofibroblasts. The latter are mainly responsible for the synthesis of a provisional ECM, thereby inducing a complex process that results in fibrosis development and scar formation [24,25,26].

In general, fibrosis markers associated with AF are an important matter of current research. It is proposed that an increased serum concentration of markers, particularly galectin-3, could be used to screen for AF [27]. However, it was not possible to confirm the difference in galectin-3 serum concentrations found in previous studies [22] in the present patient collective between paroxysmal and permanent AF.

After implantation of an LAAO device (mainly the WATCHMAN^®^ device, Boston Scientific^®^, Marlborough, MA, USA), the foreign material of the device provokes immune and inflammatory reactions that, in the end, result in a collagen deposition capsule in the form of a fibrotic scar at the material–tissue interface [28]. Fibroblasts, myofibroblasts, and macrophages have been identified as important cells in the initiation and progression of this scarring process [29]. However, ongoing fibrosis and scar formation are pivotal processes in maladaptive cardiac remodeling [29]. Galectin-3 seems to be particularly involved in both processes [29].

In our study, we only included patients who received a single foreign body in the form of a WATCHMAN^®^ LAA occluder. None of the patients had any other devices implanted 12 months prior to or during the follow-up period. The presence of additional implanted devices, such as valves, can lead to a release of galectin-3 into the bloodstream, making it difficult to accurately detect PDL in these patients [30].

Hamadanchi et al. [17] established a classification that, in particular, described the so-called Type C morphology after LAAO and identified examples featuring insufficient occlusion, such as higher rates of PDL, greater LAAO sizes and more elliptically shaped LAA orifice areas with reduced compression rates. However, the associated long-term clinical impact for thrombus formation and even occurrence of stroke is unknown. Although no such event could be detected during the 6-month follow-up, general statements cannot be formulated due to the limited size of our study group. Type C seems to carry higher levels of galectin-3 in comparison to Type A/B beyond even the 6-month interval, assuming that the acute phase after LACC is followed by chronic inflammation, which is marked by the presence of mononuclear cells (monocytes and lymphocytes) at the implant site. Furthermore, galectin-3 serves as a surrogate parameter for the intermediate type (Type B) where PDL or relevant flow within the device is unclear. If low levels of galectin-3 are present, however, it is likely that PDL is not present. In light of the already mentioned study by Alkhouli et al. [15] that identified small PDLs to be associated with a relevant risk for stroke, we speculate that this clinical impact is based on our Type B.

Regarding the exploration of additional biomarkers capable of identifying incomplete device endothelialization (IDE) and potential peri-device leakage (PDL), Xu et al. [31] conducted a study involving 55 patients. Their investigation delved into inflammatory and growth factors (IGFs), encompassing plasma levels of specific IGFs, including basic fibroblast growth factor (bFGF), platelet-derived growth factor (PDGF), stromal cell-derived factor (SDF)-1a, transforming growth factor (TGF)-β1, vascular growth factor receptor-1 (VEGF-R1), and von Willebrand factor (vWF). This analysis was conducted six months post LAAO in consecutive patients with AF. Among these six IGFs, solely the plasma level of bFGF exhibited a significant decrease in patients exhibiting IDE. Notably, employing a cut-off value of 440.52 pg/mL (with a sensitivity of 76.5% and a specificity of 78.9%), Xu et al. [31] identified lower bFGF levels as an independent factor associated with IDE. This finding was substantiated by a multivariate logistic regression model [31].

### Clinical Application of Galectin-3 for Detection of Unsuccessful LAAO

**Figure 7:**
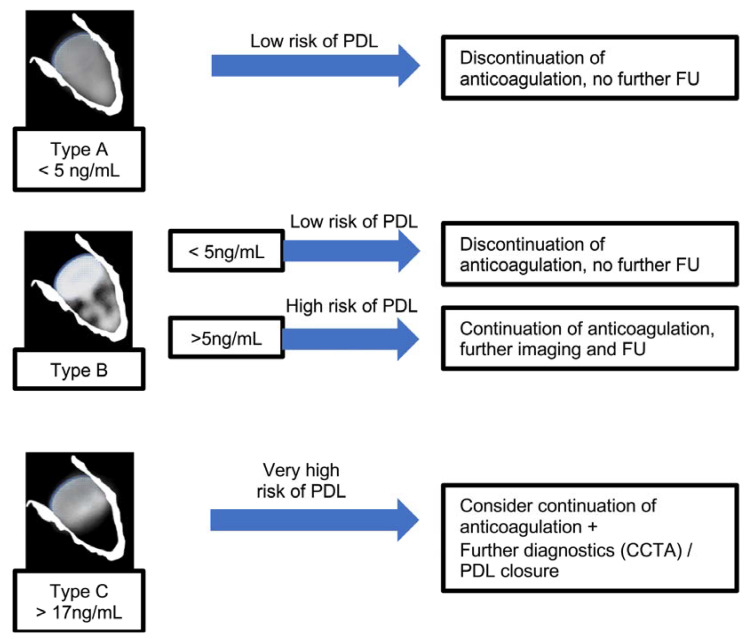
Flow chart that outlines a suggested approach to managing PDL based on echocardiographic LAAO morphology during the follow-up process.

A proposed model of a “bio-echocardiographic” classification and its clinical interpretation is shown in Figure 7, which arranges the morphologic classification and the corresponding findings of galectin-3 as a diagnostic approach for the detection of/ruling out PDL and outlines combinations for further diagnostics and/or interventional PDL occlusion. However, such a model has very restricted implications and should be reviewed and prospectively validated. The model should also be discussed in terms of the contrasting findings of the previously mentioned study by Alkhouli et al. [15]. Here, especially small leaks caused arterial embolisms. This would mean a continuation to anticoagulate all patients with at least a Type B constellation. However, leaks of relevant size were just too rare (0.5%) in the study by Alkhouli et al. [15] to statistically show a higher associated stroke rate. Therefore, further studies should focus on determining whether anticoagulation should be continued or discontinued.

Despite the findings of our study, it is important to acknowledge some limitations. Galectin-3 can be expressed not only after device implantation but also in various underlying chronic conditions, such as heart failure [32], chronic obstructive pulmonary disease (COPD) [33], and chronic kidney injury (CKI) [34]. Additionally, we acknowledge that our study lacked CT data, which could have increased the sensitivity for the detection of PDL, but this method involves the use of contrast media and radiation [10,35].

Our current study, although rather small and with the limitations mentioned above, might pave the way to further investigate galectin-3 as a promising surveillance marker after LAAO, but it needs to be clarified how galectin-3 measurements can be implemented cost-effectively in daily clinical practice since this biomarker is currently not available in routine laboratory diagnostics. In that context, point-of-care measurements, which are already available for a variety of established cardiovascular biomarkers like troponin or brain natriuretic peptide, might be an option [36]. Such a study should validate the potential not only in a large cohort of patients but also in a variety of different LAAO devices (e.g., Amplatzer Amulet^®^ (Abbott Laboratories^®^, Abbott Park, North Chicago, IL, USA) and WATCHMAN FLX^®^ (Boston Scientific^®^, Marlborough, MA, USA)).

## 4. Materials and Methods

### 4.1. Patients

Consecutive patients presenting at the University Hospital Jena during a 24-month period and undergoing LAAO were included and prospectively analyzed. In detail, the indications for LAA occlusion were a CHA_2_DS_2_VASC score ≥1 and a HASBLED score ≥3 with prior bleeding complications under therapeutic oral anticoagulation. This study was approved by the local ethics committee of the University Hospital Jena (registration number: 4816-06/16). Written informed consent was obtained from all participants prior to study participation. Eligible patients were screened at baseline. Follow-up was carried out at 45 days (45D) and 6 months (6M) after the procedure according to local standard operating procedures.

### 4.2. LAAO Procedure and Peri-Device Leak Assessment

Percutaneous LAAO procedures were performed by experienced interventional cardiologists under conscious sedation and TEE guidance as well as fluoroscopic control. Occlusion devices were introduced through the femoral vein and implanted into the LAA following a transseptal puncture. Devices were inserted in such a way that the ostium of the left atrial appendage was filled as completely as possible. Oral anticoagulation/antiplatelet therapy was resumed for 45 days following LAAO, and patients received either a dual antiplatelet therapy (aspirin 100 mg/clopidogrel 75 mg once daily) or remained on an NOAC agent or Vitamin K antagonists (VKA) with an internal normalized ratio (INR) of 2.0–3.0. A transesophageal echocardiography was performed in all patients to assess the extent of occlusion of the LAA, device-associated thrombus formation, and occluder position after 45 days and 6 months. Using color doppler interrogation, a flow between the device and LAA wall was defined as a PDL and, based on its diameter, any measurement more than 5 mm was considered as clinically significant leakage. In cases with a gap > 5 mm or device-related thrombus (DRT) formation, oral anticoagulant or NOAK use was continued. If no PDL or DRT were detected, oral anticoagulation was permanently stopped and dual antiplatelet therapy was given up to three months after implantation, and thereafter, aspirin was administered alone for up to 12 months.

### 4.3. Transesophageal Echocardiography (TEE)

TEE examinations were performed in all patients at 45 days and 6 months following the procedure using the ultrasound system IE33 or EPIQ, Philips Healthcare^®^, Andover, MA, USA. The recordings were processed using a dedicated software (Image-Arena^TM^ Version 4.6; TomTec^®^ Imaging Systems, Unterschleissheim, Germany). The detailed methodology of acquisition and analysis of the LAAs before and after the procedure was previously reported by our group [17,37].

**Figure 8:**
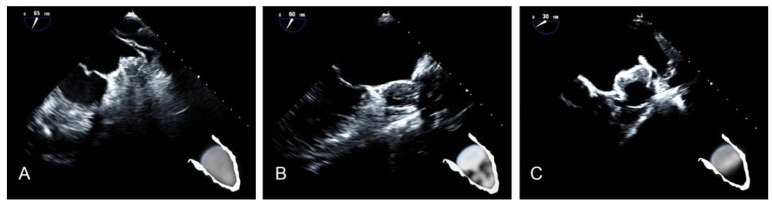
(**A**) Type A after LAAO. (**B**) Type B after LAAO. (**C**) Type C after LAAO.

Our recently proposed morphological classification of LAA occluder device appearance after implantation includes three types [17]. Type A (Figure 8A) features an entirely thrombosed device with completely homogenous echodensity on standard echo views (0–135°) [17]. Type B (Figure 8B) represents an intermediate stage with a partial flow (less than 50% of device area) within the device and another part of the device already thrombosed. It is a transitional stage with the potential of moving in both directions based on device–host interactions and amount of PDL [17]. Type C (Figure 8C) defines a partially thrombosed device in which echo-free “black” areas comprise roughly more than 50% of the total device area in at least two orthogonal views [17].

### 4.4. Serum Quantification of Galectin-3 Using ELISA Technique

Directly (within 20 min) after blood withdrawal, collection tubes were centrifuged and serum was transferred into low-binding tubes (Protein LoBind, Eppendorf^®^ AG, Hamburg, Germany), snapped frozen in liquid nitrogen, and stored at −80 °C.

Serum concentrations of galectin-3 were quantified via the ELISA technique using the following commercially available assay according to the instructions of the manufacturer: Human galectin-3 Immunoassay (Quantikine^®^ ELISA, Bio-Techne Corporation, Minneapolis, MN, USA).

### 4.5. Control Group

To provide context and comparison, serum galectin-3 levels from a database were obtained from a control group without AF and LAAO but with similar baseline characteristics.

### 4.6. Statistical Analysis

Our data were analyzed using SPSS Statistics^®^ software (version 26.0, SPSS Inc., IBM, Armonk, NY, USA). With the help of Pearson’s chi-squared test, we evaluated differences in the frequency of nominally scaled parameters. Metric variables are expressed as mean ± standard deviation; tests on differences were performed using Student’s *t*-test. Predictive values were quantified with binary logistic regression models. Correlation analyses were performed using Pearson’s test for pure metric values. Other models for prediction were generated by using receiver operating characteristic (ROC) curves, and their respective areas under the curve (AUC) values are described. The basis for the test decision was a significance level of *p* < 0.05. Cut-off values were calculated using Youden’s index derived from the ROC curve analysis for galectin-3 at 45 days, for which the patients differed in morphology (Type A/B/C). Cut-off values were determined using the maximum value for Youden’s index that was closest to 1.

## 5. Conclusions

Biochemical diagnosis and risk stratification of PDL is a very attractive but totally unknown area in interventional cardiology, and this study tried to open a new avenue for using biomarkers for early prediction of inadequate appendage occlusion. We showed that nearly all patients with PDL exhibited elevated values of galectin-3, a marker of ongoing fibrosis. Decreased or normal levels suggest successful LAA occlusion. In borderline cases, galectin-3 serves as a biochemical marker of inadequate sealing of the appendage and could be considered an additional diagnostic modality for the detection of PDL following LAAO.

## Figures and Tables

**Table 1:** Baseline characteristics of the study population.

	Study Population
	(n = 47)
Demographics	
Age [years—mean ± SD]	73.9 ± 7.1
>75 years [n (%)]	23 (49)
Male—[n (%)]	30 (64)
Female—[n (%)]	17 (36)
BMI [kg/m^2^—mean ± SD]	29.7 ± 4.9
Systolic blood pressure [mmHg—mean ± SD]	144.2 ± 22.6
Diastolic blood pressure [mmHg—mean ± SD]	74.9 ± 14.1
Heart rate [bpm—mean ± SD]	74.4 ± 14.4
**Laboratory data**	
Hemoglobin [mmol/L—mean ± SD]	9.1 ± 10.1
eGFR [mL/min/1.73 m^2^—mean ± SD]	56.5 ± 23.3
Hematocrit [%—mean ± SD]	37.8 ± 6.1
**Scores**	
CHA_2_DS_2_VASC score [mean ± SD]	3.9 ± 0.9
HAS-BLED score [mean ± SD]	5.0 ± 0.9
**Atrial Fibrillation**	
Permanent [n (%)]	26 (55)
SR at baseline and paroxysmal AF in history [n (%)]	21 (45)
**Type of anticoagulation**	
Vitamin K antagonist [n (%)]	19 (40)
Rivaroxaban [n (%)]	7 (15)
Apixaban [n (%)]	10 (21)
Dabigatran [n (%)]	5 (11)
Edoxaban [n (%)]	6 (13)
**Major bleeding complication under anticoagulation**	
Gastrointestinal [n (%)]	21 (45)
Intracranial [n (%)]	15 (32)
ENT [n (%)]	7 (15)
UT [n (%)]	2 (4)
Other [n (%)]	2 (4)
**Echocardiographic parameters**	
Left ventricular ejection fraction [%—mean ± SD]	59.1 ± 11.9
Orifice area [cm^2^—mean ± SD]	3.2 ± 1.1
Sphericity index [mean ± SD]	1.5 ± 0.21
**Procedural data**	
Occluder size [mm—mean ± SD]	26.4 ± 3.9
Compression rate [%—mean ± SD]	42.0 ± 40.9
Type of occluder	
WATCHMAN^®^ device [n (%)]	43 (92)
LAmbre device [n (%)]	4 (8)
**Comorbidities**	
Hypertension [n (%)]	44 (94)
Diabetes [n (%)]	20 (43)
Obesity [n (%)]	28 (60)
COPD [n (%)]	6 (13)
Myocardial infarction [n (%)]	12 (26)
CAD [n (%)]	20 (43)
PVD [n (%)]	9 (19)
CKD [n (%)]	42 (89)
RRT [n (%)]	2 (4)
Stroke [n (%)]	21 (45)
Heart failure [n (%)]	13 (28)

SD = standard deviation; n = absolute number; BMI = body mass index; CHA_2_DS_2_VASC = congestive heart failure, hypertension, age, diabetes mellitus, stroke/TIA/thromboembolism; HAS-BLED = hypertension, abnormal renal or liver function, stroke, bleeding, labile INR, elderly, drugs or alcohol; ENT = ear, nose, throat; UT = urinary tract; COPD = chronic obstructive pulmonary disease; eGFR = estimated glomerular filtration rate; AF = atrial fibrillation; CAD = coronary artery disease; PVD = peripheral vessel disease; CKD = chronic kidney disease; RRT = renal replacement therapy.

**Table 2:** Cut-off values for galectin-3 for detecting or ruling out Type C.

	Type C Likely Cut-Off Value (Sensitivity [%])		Type C Unlikely Cut-Off Value (Specificity [%])
45D	15.1 ng/mL (88.9)	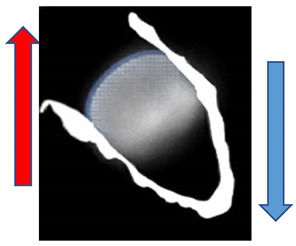	5.2 ng/mL(94.4)
6M	17.1 ng/mL (88.9)		5.2 ng/mL (94.1)

45D = 45-day follow-up; 6M = 6-month follow-up.

## Data Availability

The data presented in this study are not publicly available due to local legal restrictions on data safety.
